# Supplementary material for: An Innovative Method of Improving an Extract of Andrographis paniculata from Leaves: Its Anticancer Effect Involves the Cell Endoplasmic Reticulum
Source: Int J Mol Sci. 2025 Jan 2;26(1):344. doi: 10.3390/ijms26010344 (PMC11719592; doi:10.3390/ijms26010344)
Supplement: Supplementary file 1 [file ijms-26-00344-s001.zip › ijms-3389606-supplementary.pdf]

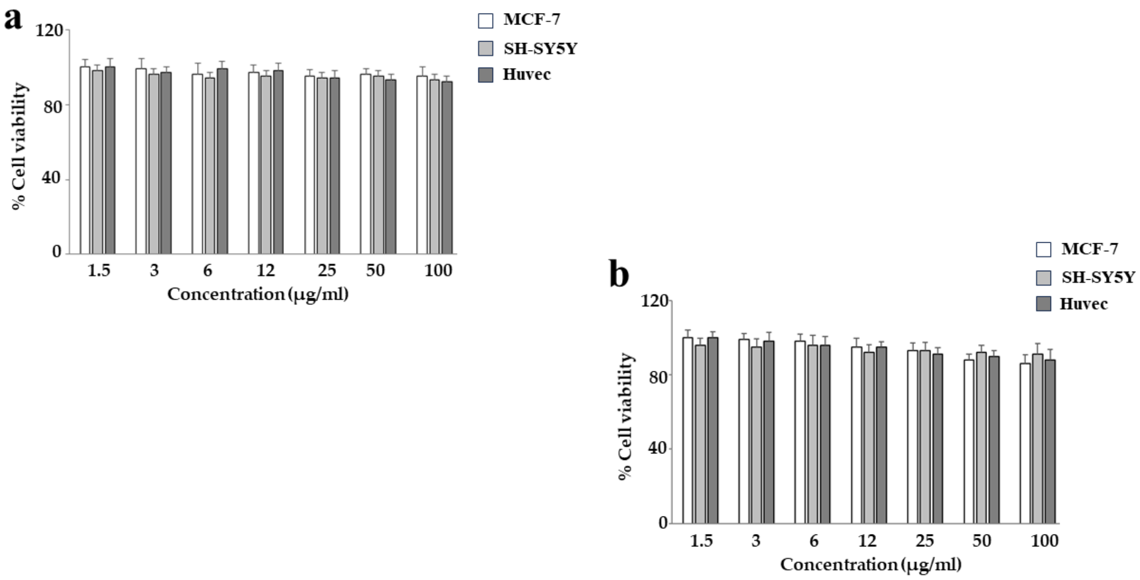

Figure S1. Effects of butanol alone, without PA and ALE, on cells. As can be seen, the effects of the vehicle butanol alone (corresponding to the concentrations used to dissolve PA and ALE, in panels (**a**,**b**), respectively) were represented. Three independent experiments were carried out, and the values are expressed as the mean ± SD.

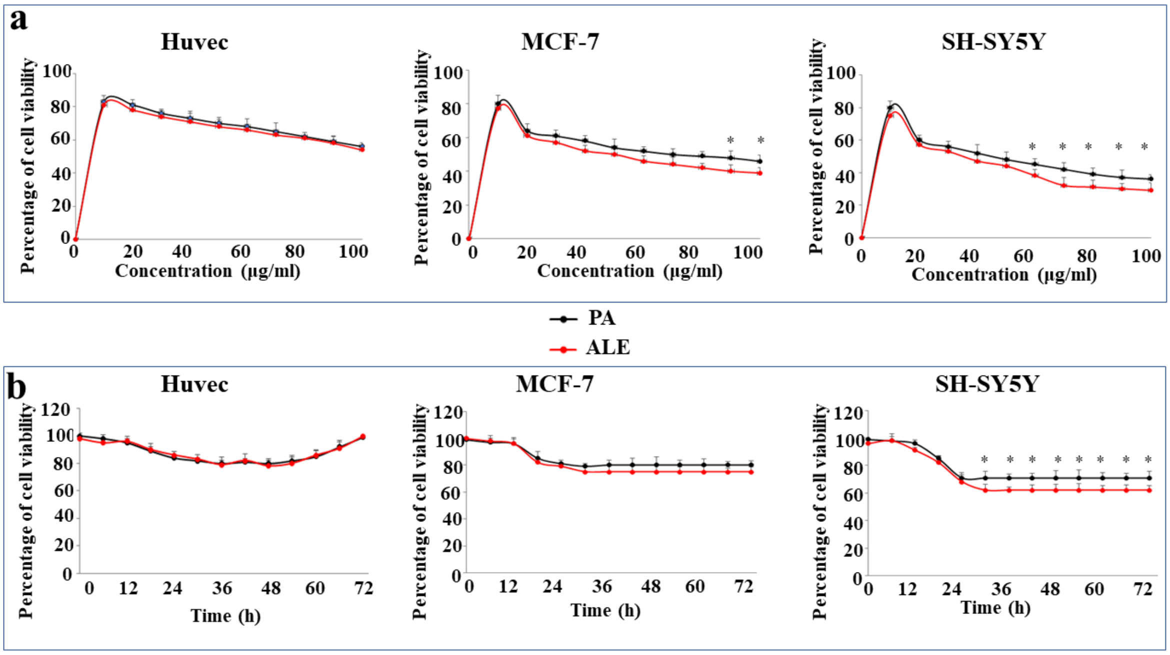

Figure S2. Dose–response curves and time–response curves. In Panel (**a**), the dose–response curves following treatment with PA and ALE are shown in the three cell lines HUVEC, MCF-7, and SH-SY5Y (**left**, **center**, and **right**, respectively). With the same arrangement, Panel (**b**) highlights time–response curves. Three independent experiments were carried out, and the values are expressed as the mean ± SD. * denotes $p < 0.05$ vs. the respective ALE. A Tukey–Kramer comparison test followed the analysis of Variance (ANOVA).
