# Supplementary figures and images for: An Innovative Method of Improving an Extract of Andrographis paniculata from Leaves: Its Anticancer Effect Involves the Cell Endoplasmic Reticulum
Source: Int J Mol Sci. 2025 Jan 2;26(1):344. doi: 10.3390/ijms26010344 (PMC11719592; doi:10.3390/ijms26010344)

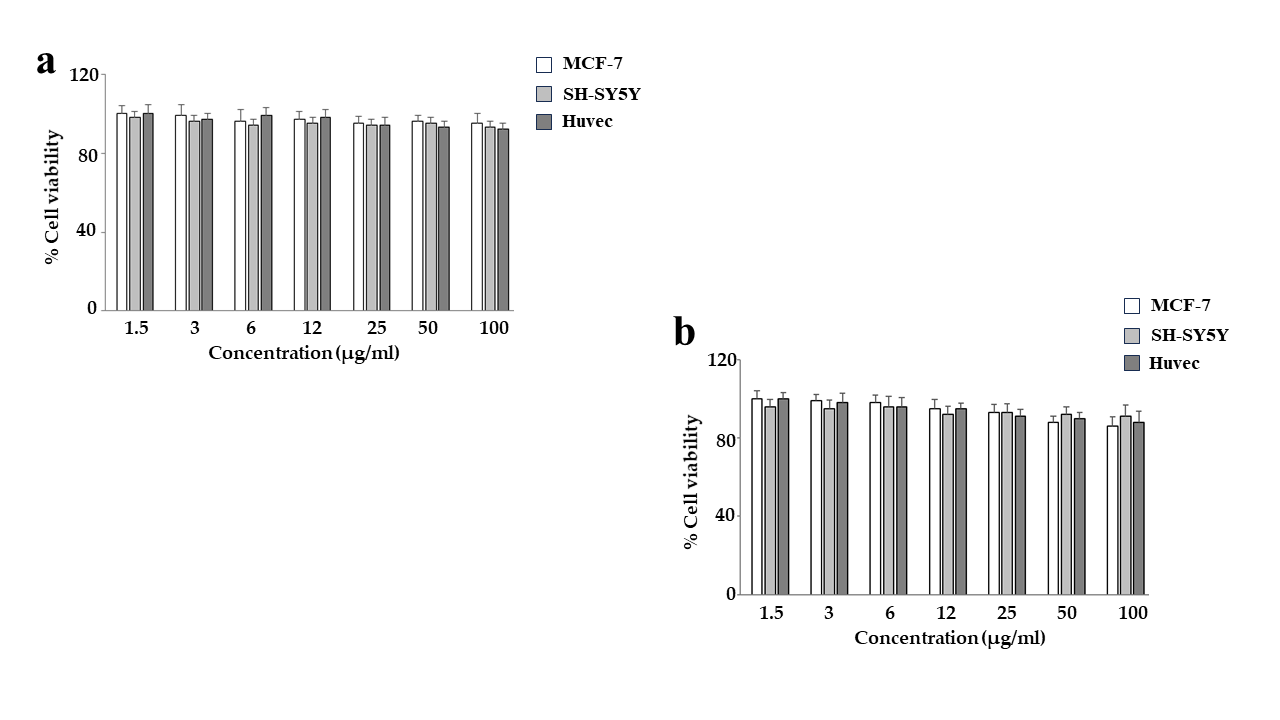

Supplement: Supplementary file 1 [file ijms-26-00344-s001.zip › supplementary Figure S1.tif]

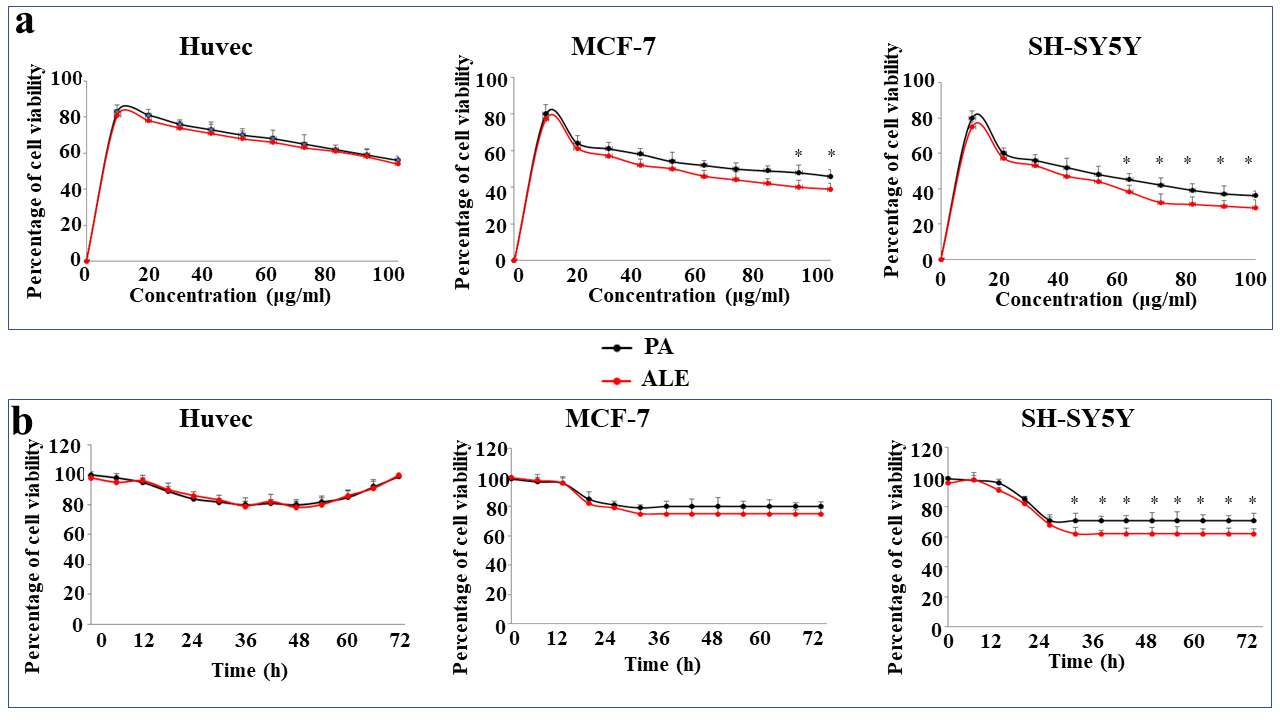

Supplement: Supplementary file 1 [file ijms-26-00344-s001.zip › Supplementary Figure S2.tif]
